# Effect of Different Aging Methods on Surface Microhardness and Roughness of Anterior Resin Composites: An In Vitro Study

**DOI:** 10.3390/ma18204684

**Published:** 2025-10-13

**Authors:** Cansu Dağdelen Ahısha, Mine Betül Üçtaşlı

**Affiliations:** Department of Restorative Dentistry, Faculty of Dentistry, Gazi University, Ankara 06490, Türkiye; uctasli@gazi.edu.tr

**Keywords:** resin composite, microhardness, water storage, thermal cycling, surface roughness

## Abstract

The surface properties of composite resin restorative materials are critical for the esthetics and longevity of restorations. This in vitro study evaluated the microhardness change and surface roughness change in four resin composites recommended for anterior restorations after two aging simulations: thermal cycling (10,000 cycles) and one year of water storage. Ten specimens (*n* = 10) were prepared for each material. After baseline measurements, samples were subjected to one of the aging procedures, and surface properties were reassessed. For microhardness change (∆H), significant differences were observed among materials under both thermal cycling (*p* = 0.001) and water storage (*p* = 0.001). Omnichroma–thermal cycling showed a greater decrease than G-ænial Anterior (*p* = 0.028) and Clearfil Majesty ES-2 (*p* = 0.001), while Optishade–thermal cycling decreased more than Clearfil Majesty ES-2 (*p* = 0.015). In water storage, Omnichroma exhibited a greater decrease than Optishade (*p* = 0.042) and Clearfil Majesty ES-2 (*p* = 0.001), and G-ænial Anterior decreased more than Clearfil Majesty ES-2 (*p* = 0.026). Optishade and Clearfil Majesty ES-2 showed significantly greater decreases after thermal cycling than water storage, while Omnichroma and G-ænial Anterior showed no difference. For the change in surface roughness (∆R), significant differences were also found (*p* = 0.001). In thermal cycling, Optishade exhibited the lowest increase, while G-ænial Anterior showed the highest. In water storage, G-ænial Anterior again had the highest increase, significantly greater than all others (*p* = 0.001). For all materials, ∆R values were significantly higher after thermal cycling compared with those in water storage (*p* = 0.001). These results demonstrate that both composite type and aging method influence long-term surface properties. Overall, thermal cycling exerted more detrimental effects than water storage.

## 1. Introduction

The increasing demand for cosmetic outcomes in dental applications has led to a growing interest in direct resin composite restorations. These materials are preferred due to their enhanced esthetics, improved physical properties, and ease of application [1]. The surface properties of composite resin restorative materials play a significant role in the esthetics of teeth restored with these materials [2,3,4].

The physical and mechanical properties of composite resin restorative materials are influenced by their chemical composition, organic and inorganic structure, and type of filler [5,6]. The surface microhardness of composite resin restorative materials is closely related to their mechanical properties. Various factors related to the composition of these materials such as the type of monomer, filler size, and filler content can affect their mechanical performance [7,8]. A low surface microhardness value may lead to plaque accumulation [9,10].

Rough surfaces on restorations made with direct composite resin restorative materials may increase plaque retention and lead to various clinical problems [11]. Increased plaque accumulation can result in restoration staining, gingivitis, and a higher risk of recurrent caries. According to the literature, the surface roughness of composite resin restorative materials is affected by various factors, such as the type, shape, size, and distribution of filler particles; the type of resin matrix; and the bonding efficiency between the filler and matrix [12].

Dental restorations in the oral environment are exposed to several factors such as moisture, thermal changes, and chemical and biological agents in saliva. In the literature, it is stated that aging procedures can be performed using mechanical, thermal, or chemical methods, as well as combinations of these approaches [13]. While thermal cycling is a widely used artificial aging method to simulate the physiological aging of restorative materials, water storage is a much more time-consuming approach compared to thermal cycling [13]. These variable conditions can alter the surface properties of restorative materials. In composite resin restorative materials, the primary reason for these changes is the diffusion of unreacted resin monomer molecules and ions, as well as the polymeric matrix that makes up the structure [14].

Water molecules interacting with composite resin restorative materials typically cause the material to swell due to moisture absorption and the polymer network to soften [15]. Over time, this can compromise the restoration’s mechanical properties and resistance to wear [15].

Alshali et al. [16] investigated the water sorption and solubility of conventional and bulk-fill resin composites after one year of storage in water and artificial saliva. They reported that both materials exhibited these effects. Islam et al. [17] demonstrated in their study comparing the physical and optical stability of resin composites with different filler characteristics that the materials showed significant differences in Vickers hardness, water sorption (with composite blocks stored in deionized water at 37 °C for 7 days in an incubator), solubility, and color stability.

The literature clearly shows a lack of studies directly comparing the resin composite restorative materials evaluated in this study under two different aging methods that simulate clinical conditions. This gap hinders the comparative assessment of how different materials withstand mechanical and optical changes during aging.

This study aims to evaluate the surface properties, specifically microhardness and surface roughness, of composite resin restorative materials recommended for anterior use after two different aging protocols (10,000 thermal cycles/1-year water storage) that simulate one year of intraoral aging.

This study was conducted under the assumption of two main null hypotheses. The first hypothesis states that there is no significant difference in the microhardness of composite resin restorative materials, either among the different materials or between the two aging methods (10,000 thermal cycles and 1-year water storage). The second hypothesis states that there is no significant difference in the surface roughness of composite resin restorative materials, either among the different materials or between the two aging methods (10,000 thermal cycles and 1-year water storage).

## 2. Materials and Methods

### 2.1. Sample Preparation

Prior to group allocation, a power analysis was conducted to determine the appropriate sample size. Based on an effect size of 0.5, a significance level of α = 0.05, and a desired statistical power (1–β) of 0.80, a minimum of eight specimens per group was required. To accommodate potential outliers, ten specimens were allocated to each group.

This study included four different composite resin restorative materials recommended for anterior restorations: Optishade (Kerr Corporation, Brea, CA, USA), Clearfil Majesty ES-2 (Kuraray Medical Inc., Okayama, Japan), Omnichroma (Tokuyama, Yamaguchi Prefecture, Japan), and G-ænialAnterior (GC Corporation, Tokyo, Japan). The technical properties of the composite resin restorative materials used in the study are shown in Table 1. For each material, 10 specimens were prepared for surface roughness and microhardness measurements (*n* = 10) (Table 2).

Plexiglass molds with a diameter of 4 mm and a depth of 2 mm were used for specimen preparation. The molds were two-piece systems, allowing easy removal of the specimens without damage. During specimen preparation, the restorative materials were placed into the molds; a transparent polyester (PET Mylar) matrix strip of 0.05 mm thickness was first applied, followed by a glass slide (laminate), which was gently pressed against both surfaces. Polymerization was then performed for 20 s using an LED light-curing unit (D-Light Pro, GC, Tokyo, Japan) with a wavelength of 430–480 nm and a light intensity of 1200 mW/cm^2^, in accordance with the manufacturer’s instructions.

The bottom surfaces of the specimens were marked with a scalpel. After preparation, the specimens were stored in distilled water at 37 °C for 24 h in an incubator. The polishing procedure was performed using polishing discs (Sof-Lex-XT (Aluminum oxide abrasives), 3M ESPE, St. Paul, MN, USA) in sequence from coarse to superfine grit, and an angled handpiece was used for polishing. The discs and their particle sizes were as follows: Coarse 92–98 μm, Medium 25–29 μm, Fine 16–21 μm, and Superfine 2–5 μm. Each disc was applied to the surface for 10 s at 10,000 rpm, followed by rinsing with water for 10 s and drying with light air for 5 s.

### 2.2. Aging Methods

After initial surface roughness and microhardness measurements, the specimens were divided into two groups to undergo different aging protocols. In this study, only thermal cycling and water storage methods were evaluated, as these two approaches are both commonly used and highly feasible for simulating clinical conditions in a laboratory setting.

Thermal Cycling: Half of the specimens (*n* = 10 per material) were subjected to thermal cycling to simulate 1 year of clinical aging. The samples were placed in the basket compartment of a thermal cycling device (Thermocycler, SD Mechatronik, Feldkirchen-Westerham, Germany) and exposed to 10,000 cycles between water baths at 5 °C and 55 °C. Each immersion lasted 20 s, with a 10-s dwell time between cycles. Following the thermal cycling procedure, surface roughness and microhardness measurements were performed (T_1_).

Water Storage: The other half of the specimens (*n* = 10 per material) were stored in distilled water at 37 °C for 1 year in an incubator to simulate long-term aging. The distilled water was replaced weekly, and no visible microbial growth was observed. The pH of the water was monitored periodically and maintained at neutral. Surface roughness and microhardness measurements were performed after completion of the water storage procedure (T_2_).

### 2.3. Vickers Microhardness Measurement

The microhardness values of the top surfaces of the specimens were measured using a Vickers microhardness tester (HMV-700 Microhardness Tester, Shimadzu, Kyoto, Japan). During the test, a load of 490 N was applied for 10 s, and each sample was measured three times. The average of these three measurements was recorded as the microhardness number (VHN) of the material. Measurements were performed at three time points: 24 h after sample preparation (T_0_), after 10,000 thermal cycles (T_1_), and after 1 year of water storage (T_2_).

### 2.4. Surface Roughness Measurement

Surface roughness of the samples was measured using a profilometer (Surftest SJ-301; Mitutoyo, Aurora, IL, USA) by selecting an area of 100 × 100 μm^2^. Each measurement was repeated three times, rotating the sample clockwise around its center, and the average value was calculated. Measurements were taken at three time points: 24 h after sample preparation (T_0_), after 10,000 thermal cycles (T_1_), and after 1 year of water storage (T_2_).

## 3. Statistical Analysis

The data obtained in this study were analyzed using IBM SPSS Statistics 22. The normality of the data distribution was assessed using the Kolmogorov–Smirnov and Shapiro–Wilk tests, and it was determined that the parameters followed a normal distribution. To evaluate the effect of composite resin restorative material and aging method on changes in surface microhardness and surface roughness, Two-Way ANOVA was used. A *p*-value of <0.05 was considered statistically significant.

## 4. Results

### 4.1. Microhardness Results 

**Figure 1:**
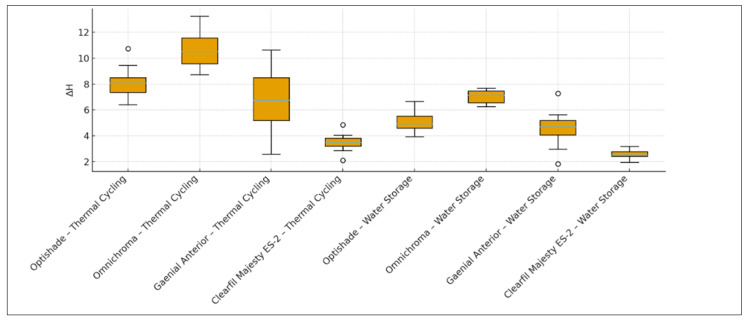
Evaluation of Microhardness Changes According to Composite Resin Restorative Materials and Aging Methods.

When thermal cycling was applied, there was a statistically significant difference in microhardness change values (∆H) among the composite resin restorative materials (*p* = 0.001; *p* < 0.05) (Table 3 and Table 4, Figure 1). The microhardness change (decrease) in the Omnichroma–thermal cycling group was significantly higher than in the G-aenial Anterior–thermal cycling group (*p* = 0.028) and the Clearfil Majesty ES-2–thermal cycling group (*p* = 0.001) (*p* < 0.05). The ∆H value (decrease) of the Optishade–thermal cycling group was also significantly higher than that of the Clearfil Majesty ES-2–thermal cycling group (*p* = 0.015) (*p* < 00.5). There were no statistically significant differences in ∆H values between the other groups (*p* > 0.05) (Table 3 and Table 4 and Figure 1).

When water storage was applied, there was also a statistically significant difference in ∆H values among the composite resin restorative materials (*p* = 0.001; *p* < 0.05) (Table 3 and Table 4 and Figure 1). The ∆H value (decrease) of the Omnichroma–water storage group was significantly higher than that of the Optishade–water storage (*p* = 0.042) and Clearfil Majesty ES-2–water storage (*p* = 0.001) groups (*p* < 0.05). The ∆H value (decrease) in the G-aenial Anterior–water storage group was also significantly higher than that of the Clearfil Majesty ES-2–water storage group (*p* = 0.026) (*p* < 0.05). There were no statistically significant differences in ∆H values among the other groups (*p* > 0.05) (Table 3 and Table 4 and Figure 1).

For the Optishade (*p* = 0.003; *p* < 0.05) and Clearfil Majesty ES-2 (*p* = 0.006; *p* < 0.05) composite resin restorative materials, the ∆H value after thermal cycling was significantly higher than after water storage (Table 3 and Table 4, Figure 1).

For the Omnichroma (*p* = 0.106; *p* > 0.05) and G-aenial Anterior (*p* = 0.582; *p* > 0.05) composite resin material, there was no statistically significant difference in ∆H values between thermal cycling and water storage (Table 3 and Table 4, Figure 1).

### 4.2. Surface Roughness Results

**Figure 2:**
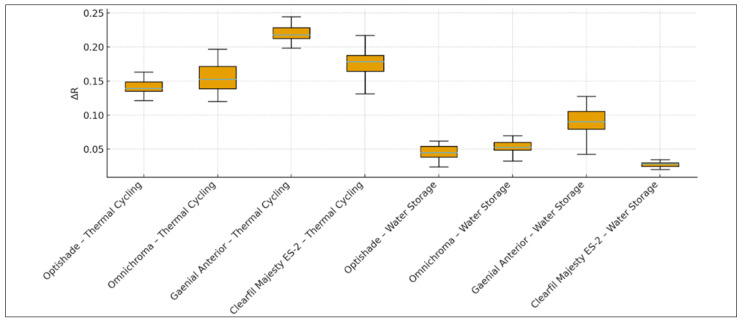
Evaluation of Roughness Changes According to Composite Resin Restorative Material and Aging Methods.

When thermal cycling was applied, there was a statistically significant difference in surface roughness change values (∆R) among the composite resin restorative materials (*p* = 0.001; *p* < 0.05) (Figure 2, Table 5 and Table 6). The ∆R value (increase) of the Optishade–thermal cycling group was significantly lower than that of the Omnichroma–thermal cycling group (*p* = 0.014), G-aenial Anterior–thermal cycling group (*p* = 0.001), and Clearfil Majesty ES-2–thermal cycling group (*p* = 0.009) (*p* < 0.05). The ∆R value (increase) of the G-aenial Anterior–thermal cycling group was significantly higher than that of the Optishade–thermal cycling group (*p* = 0.001), Omnichroma–thermal cycling group (*p* = 0.001), and Clearfil Majesty ES-2–thermal cycling group (*p* = 0.001) (*p* < 0.05). There was no statistically significant difference between the Omnichroma and Clearfil Majesty ES-2 thermal cycling groups (*p* > 0.05) (Figure 2, Table 5 and Table 6).

When water storage was applied, a statistically significant difference in ∆R values was observed among the composite resin restorative materials (*p* = 0.001; *p* < 0.05) (Figure 2, Table 5 and Table 6). The ∆R value (increase) of the G-aenial Anterior–water storage group was significantly higher than those of the Optishade–water storage group (*p* = 0.001), Omnichroma–water storage group (*p* = 0.001), and Clearfil Majesty ES-2–water storage group (*p* = 0.001) (*p* < 0.05). The ∆R value (increase) of the Clearfil Majesty ES-2–water storage group was significantly higher than that of the Optishade–water storage group (*p* = 0.040; *p* < 0.05) (Figure 2, Table 5 and Table 6). No statistically significant differences were found between other groups (*p* > 0.05) (Figure 2, Table 5 and Table 6).

For Optishade composite resin restorative material, the amount of ∆R (increase) with the thermal cycling method was statistically significantly higher than with the water storage method (*p* = 0.001; *p* < 0.05) (Figure 2, Table 5 and Table 6).

For Omnichroma composite resin restorative material, the amount of ∆R (increase) with the thermal cycling method was statistically significantly higher than with the water storage method (*p* = 0.001; *p* < 0.05) (Figure 2, Table 5 and Table 6).

For G-aenial Anterior composite resin restorative material, the amount of ∆R (increase) with the thermal cycling method was statistically significantly higher than with the water storage method (*p* = 0.001; *p* < 0.05) (Figure 2, Table 5 and Table 6).

For Clearfil Majesty ES-2 composite resin restorative material, the amount of ∆R (increase) with the thermal cycling method was statistically significantly higher than with the water storage method (*p* = 0.001; *p* < 0.05) (Figure 2, Table 5 and Table 6).

## 5. Discussion

This study aimed to evaluate the surface properties, specifically microhardness and surface roughness, of composite resin restorative materials recommended for the anterior region after simulating one year of aging in the oral environment using two different aging protocols (10,000 thermal cycles and 1-year water storage). This study was conducted under the assumption of two main null hypotheses. The first null hypothesis stated that there would be no significant difference in the microhardness of composite resin restorative materials, either among the different materials or between the two aging methods. This hypothesis was partially rejected. Statistically significant differences in microhardness were observed among the materials after both thermal cycling and water storage (*p* = 0.001; *p* < 0.05). Furthermore, comparisons between the two aging methods revealed that microhardness changes varied depending on the material. While Omnichroma and G-aenial Anterior showed no significant differences between thermal cycling and water storage, Optishade and Clearfil Majesty ES-2 exhibited significantly higher microhardness reductions after thermal cycling (*p* < 0.05).

The second null hypothesis stated that there would be no significant difference in the surface roughness of composite resin restorative materials, either among the different materials or between the two aging methods. This hypothesis was rejected. Statistically significant differences in surface roughness were found among the materials after both thermal cycling and water storage (*p* = 0.001; *p* < 0.05). Additionally, for all tested composites, the increase in surface roughness caused by thermal cycling was significantly higher compared to water storage (*p* = 0.001; *p* < 0.05).

Surface roughness refers to the fine irregularities of the surface layer originating from the production process and material properties of the material [18]. The smoothness of the surface improves the esthetic appearance of composite resin restorative materials. High surface roughness values can result in plaque accumulation, recurrent caries, and problems in color stability [19]. As the size of the filler particles within the restorative material decreases, better surface quality is achieved [20]. Nanohybrid composite resin restorative materials with filler sizes of 0.4–1 μm are widely used due to their superior physical properties and good polishability [21]. According to the literature, surface roughness values above 0.2 µm have been reported to increase plaque retention [22]. The initial surface roughness values of the materials evaluated in this study, and the surface roughness values of all composite resin restorative materials after aging in water storage, are clinically acceptable. However, the surface roughness of all composite resin restorative materials after aging by thermocycling exceeds clinically acceptable levels.

Surface microhardness provides information about a material’s deformation [10]. Surface microhardness indicates the resistance of the material to indentation and is important for the stability of the restoration [23]. In the literature, Vickers and Knoop hardness tests are used to evaluate the surface hardness of dental materials [24]. In this study, the Vickers hardness test was used to assess the surface microhardness of composite resin restorative materials.

Composite resin restorative materials comprise an organic matrix, inorganic filler particles, and an intermediate phase [25]. In other words, the microhardness of composite resins can vary depending on the size and proportion of filler particles, the degree of monomer conversion, and the type of inorganic filler used [26]. According to the literature, as filler particle size decreases and filler content increases, the wear resistance of composite resin restorative materials improves [25,27,28,29]. Increasing filler content also increases the surface hardness of composite resin restorative materials [30,31]. In the current study, considering the filler content reported by the manufacturers, a moderate correlation was observed between microhardness and filler content. Optishade, with a filler content of 81% by weight, showed the highest microhardness values. However, in contrast to the literature, Omnichroma, which has the second-highest filler content among the evaluated composite resin restorative materials, showed the lowest surface microhardness values. Nezir et al. [32] reported in their study, which evaluated the surface properties of monochromatic universal composite resins following exposure to a detox solution, that Omnichroma exhibited low microhardness values. They suggested that this could be attributed to differences in Omnichroma’s chemical composition, variations in filler particle size and content, and structural differences in the supra-nano spherical fillers present in its composition. Similarly, Dumitrescu et al. [33] examined the effect of 10-day exposure of G-ænial A’CHORD and Omnichroma resin composites to red wine, coffee, and Coca-Cola on their microhardness using the Vickers method. They found that Omnichroma experienced a more pronounced softening. This change in microhardness can be explained by the greater impact of degradation at the resin–filler interface on the resin matrix and the inorganic filler particles of the composite resin restorative material [34].

Aromatic groups in the structure of Bis-GMA provide a polymeric network with higher surface hardness [35]. Additionally, the use of bifunctional monomers such as TEGDMA increases surface hardness values [36]. Supporting the literature, the Optishade group containing both Bis-GMA and TEGDMA showed statistically significantly higher microhardness values at T_0_, T_1_, and T_2_ compared to other groups.

While the physiological temperature of the oral environment remains stable, it ranges between 36.3–37.1 °C in males and 36.5–37.3 °C in females [37]. The oral environment is dynamic and experiences temperature changes due to food consumption. Moreover, it is influenced by multiple factors such as food residues, bacterial activity, pH variations, and enzymatic activity (e.g., amylases) [37]. According to the literature, approximately 50.000 thermal cycles occur in the oral environment annually [38]. The physiological events causing temperature changes such as eating, drinking, and breathing are simulated as thermal cycles [39]. According to the literature, temperature changes between 5 °C and 55 °C are generally preferred during thermal cycling simulation [40,41,42].

For Optishade and Clearfil Majesty ES-2 composite resin restorative materials, the ∆S amount after thermal cycling was statistically significantly higher than after water storage (*p*: 0.003; *p* < 0.05), while no significant difference was found for Omnichroma and G-ænial Anterior composites (*p*: 0.106; *p* > 0.05).

Oliveira et al. [36] and Tuncer et al. [43] reported that thermal cycling hydrolyzes the coupling agents bonding the fillers in the composite resin restorative material matrix, thereby increasing surface roughness. According to the results of this study, surface roughness increased compared to baseline after both aging techniques. Additionally, for all composite resin restorative materials, the amount of surface roughness increase (∆R) after thermal cycling was statistically significantly higher than that after water storage (*p*: 0.001; *p* < 0.05).

Coelho-De-Souza et al. [44] reported that water storage significantly affects restoration quality. Exposure of composite resin restorative materials to water weakens the polymer matrix and damages its structure [45]. According to Hahnel et al. [46], the degradation of surface properties after water storage is attributed to water absorption, causing polymer network breakdown, resin matrix degradation, and hydrolysis at the filler–matrix interface. Supporting the literature, the data from this study show that the surface roughness and microhardness values of all evaluated composite resin restorative materials decreased after both thermal cycling and water storage compared to baseline. The microhardness amount decrease (∆H) in the Omnichroma thermal cycling group was higher than that of the G-ænial Anterior and Clearfil Majesty ES-2 thermal cycling groups. The microhardness decrease in the Optishade thermal cycling group was significantly higher than in the Clearfil Majesty ES-2 thermal cycling group. The microhardness decrease in the Omnichroma water storage group was higher than in the Optishade and Clearfil Majesty ES-2 water storage groups. The microhardness decrease in the G-ænial Anterior water storage group was significantly higher than in the Clearfil Majesty ES-2 water storage group. The surface roughness increase (∆R) in the Optishade thermal cycling group was significantly lower than in the Omnichroma, G-ænial Anterior, and Clearfil Majesty ES-2 thermal cycling groups. The surface roughness increase in the G-ænial Anterior thermal cycling group was higher than in the other thermal cycling groups. There was no statistically significant difference between the Omnichroma and Clearfil Majesty ES-2 thermal cycling groups. The surface roughness increase (∆R) in the G-ænial Anterior water storage group was significantly higher than in the Optishade, Omnichroma, and Clearfil Majesty ES-2 water storage groups. The surface roughness increase in the Clearfil Majesty ES-2 water storage group was higher than in the Optishade water storage group.

Despite simulating oral conditions, this in vitro study has limitations. Only two aging methods were applied, and other common protocols (e.g., pH cycling, brushing simulation) were not included, which should be considered a limitation. The oral environment is dynamic and influenced by multiple factors that cannot be fully replicated in vitro. Additionally, biofilm formation, mechanical wear, and pH variations were not simulated in this study, which further limits direct extrapolation to clinical conditions. Nevertheless, the results provide valuable information on the long-term surface properties of anterior composite resin restorative materials, and this information is important in material selection for clinical practice. Optishade and Clearfil Majesty ES-2 may be more suitable for anterior restorations based on surface property performance after aging.

## 6. Conclusions

Within the limitations of this in vitro study:

When thermal cycling was applied, there was a statistically significant difference in microhardness change values (∆H) among the composite resin restorative materials (*p* = 0.001; *p* < 0.05). The decrease in microhardness in the Omnichroma–thermal cycling group was significantly higher than in the G-aenial Anterior–thermal cycling group (*p* = 0.028) and the Clearfil Majesty ES-2–thermal cycling group (*p* = 0.001). The ∆H decrease in the Optishade–thermal cycling group was also significantly higher than in the Clearfil Majesty ES-2–thermal cycling group (*p* = 0.015).

For Optishade (*p* = 0.003) and Clearfil Majesty ES-2 (*p* = 0.006), the ∆H decrease after thermal cycling was significantly greater than after water storage.

For Omnichroma (*p* = 0.106) and G-aenial Anterior (*p* = 0.582), there was no significant difference in ∆H values between thermal cycling and water storage.

When thermal cycling was applied, there was a statistically significant difference in surface roughness change values (∆R) among the materials (*p* = 0.001; *p* < 0.05).

The ∆R increase in the Optishade–thermal cycling group was significantly lower than that in Omnichroma (*p* = 0.014), G-aenial Anterior (*p* = 0.001), and Clearfil Majesty ES-2 (*p* = 0.009).

The ∆R increase in the G-aenial Anterior–thermal cycling group was significantly higher than that in Optishade (*p* = 0.001), Omnichroma (*p* = 0.001), and Clearfil Majesty ES-2 (*p* = 0.001).

No significant difference was found between Omnichroma and Clearfil Majesty ES-2 (*p* > 0.05).

With water storage, G-aenial Anterior showed significantly higher ∆R increases than Optishade, Omnichroma, and Clearfil Majesty ES-2 (all *p* = 0.001). Clearfil Majesty ES-2 also had a significantly higher ∆R increase than Optishade (*p* = 0.040).

Across all materials, thermal cycling generally caused significantly greater increases in surface roughness (∆R) compared to water storage (all *p* = 0.001).

Clinical relevance: These findings highlight that surface properties of composite resins differ considerably under different aging conditions. Optishade and Clearfil Majesty ES-2 demonstrated relatively better performance regarding microhardness and surface roughness, suggesting that they may be more suitable for anterior restorations. Nevertheless, aging remains a critical factor influencing material longevity. Further well-designed in vitro and in vivo studies are needed to comprehensively evaluate the long-term effects of aging, including biofilm formation, pH changes, and mechanical wear.

## Figures and Tables

**Table 1:** Materials, manufacturers, composition, and filler content (^1^—Bis-GMA: Bisphenol A-Glycidyl Methacrylate, ^2^—Bis-EMA: Bisphenol A Ethoxylated Dimethacrylate, ^3^—TEGDMA: Triethylene Glycol Dimethacrylate, ^4^—UDMA: Urethane Dimethacrylate).

Material	Manufacturer	Matrix	Filler/Particle Size	Filler Content (%wt-%vol)	Lot Number
Optishade (nanohybrid)	Kerr Corporation, CA, USA	Bis-GMA ^1^, Bis-EMA ^2^, TEGDMA ^3^	Spherical silica and zirconia/5–400 nm	81%-64.5%	8,247,234
Clearfil Majesty ES-2 (nano filler)	Kuraray Medical Inc., Okayama, Japan	Bis-GMA^1^	Silanated barium glass filler/0.37–1.5 μm	78%-66%	320,084
Omnichroma (supra-nano spherical)	Tokuyama, Yamaguchi Prefecture, Japan	UDMA^4^, TEGDMA ^3^	Supra-nano spherical fillers (260 nm spherical SiO_2_-ZrO_2_)/2.6 μm	79%-68%	188M3
G-ænial Anterior (hybrid)	GC Corporation, Tokyo, Japan	UDMA ^4^	Pre-polymerized fillers, silica, strontium, and lanthanide fluo-silica/850 nm	63% (vol)	2,101,181, 2,009,041

**Table 2:** Grouping of the samples.

Group	Composite Resin Restorative Material—Aging Method
1	Optishade—Thermal Cycling
2	Omnichroma—Thermal Cycling
3	G-ænial Anterior—Thermal Cycling
4	Clearfil Majesty ES-2—Thermal Cycling
5	Optishade—Water Storage
6	Omnichroma—Water Storage
7	G-ænial Anterior—Water Storage
8	Clearfil Majesty ES-2—Water Storage

**Table 3:** Evaluation of Microhardness Number Changes According to Composite Resin Restorative Materials and Aging Methods.

	∆H (Difference in VHN)			
	Thermal Cycling Aging	Water Immersion Aging		
Composite	Mean ± SD	Mean ± SD	Mean Difference (95%CI)	*p*
**Optishade**	7.39 ± 1.43	4.36 ± 0.82	3.03 (1.32–4.73)	0.003 *
**Omnichroma**	9.27 ± 2.51	6.93 ± 0.69	2.33 (−0.74–5.41)	0.106
**G-ænial Anterior**	5.59 ± 2.19	4.75 ± 2.47	0.85 (−2.55–4.25)	0.582
**Clearfil Majesty ES-2**	3.37 ± 0.60	1.95 ± 0.60	1.41 (0.53–2.29)	0.006
***p***	0.001 *	0.001 *		

Two-way ANOVA Test * *p* < 0.05.

**Table 4:** Microhardness Values (VHN) According to Composite Resin Restorative Materials and Aging Methods.

	VHN	
	Thermal Cycling Aging	Water Immersion Aging
Composite	Initial/Final Measurement	Initial/Final Measurement
**Optishade**	92.54/85.16	92.36/88
**Omnichroma**	72.18/62.92	72.29/65.36
**G-ænial Anterior**	85.13/79.54	85.79/81.04
**Clearfil Majesty ES-2**	87.37/84.0	87.08/85.12

**Table 5:** Evaluation of Roughness Changes According to Composite Resin Restorative Material and Aging Methods.

	∆R (Difference in Ra Values)			
	Thermal Cycling Aging	Water Immersion Aging		
Composite	Mean ± SD	Mean ± SD	Mean Difference (95%CI)	*p*
**Optishade**	0.13 ± 0.01	0.02 ± 0.02	0.11 (0.09–0.13)	0.001 *
**Omnichroma**	0.16 ± 0.01	0.03 ± 0.01	0.13 (0.11–0.15)	0.001 *
**G-ænial Anterior**	0.22 ± 0,02	0.09 ± 0.02	0.13 (0.10–0.15)	0.001 *
**Clearfil Majesty ES-2**	0.17 ± 0.01	0.05 ± 0.01	0.12 (0.10–0.14)	0.001 *
***p***	0.001 *	0.001 *		

Two-way ANOVA Test * *p* < 0.05.

**Table 6:** Surface Roughness Values According to Composite Resin Restorative Materials and Aging Methods.

	Surface Roughness (Ra) Values	
	Thermal Cycling Aging	Water Immersion Aging
Composite	Initial/Final Measurement	Initial/Final Measurement
**Optishade**	0.086/0.218	0.086/0.10
**Omnichroma**	0.067/0.23	0.066/0.098
**G-ænial Anterior**	0.072/0.287	0.072/0.162
**Clearfil Majesty ES-2**	0.078/0.244	0.077/0.123

## Data Availability

The original contributions presented in this study are included in the article. Further inquiries can be directed to the corresponding author.

## Abbreviations

Bis-GMA	Bisphenol A-Glycidyl Methacrylate
Bis-EMA	Bisphenol A Ethoxylated Dimethacrylate
UDMA	Urethane Dimethacrylate
TEGDMA	Triethylene Glycol Dimethacrylate
µm	Micrometer
nm	Nanometer
vol	Volume
T_0_	Measurement taken 24 h after sample preparation
T_1_	Measurement taken after 10,000 thermal cycles
T_2_	Measurement taken after 1 year of water storage
∆R	Surface roughness change values
∆H	Microhardness change values
VHN	Microhardness Values
